# Blood Pressure in Different Dementia Disorders, Mild Cognitive Impairment, and Subjective Cognitive Decline

**DOI:** 10.3389/fnagi.2020.00257

**Published:** 2020-08-31

**Authors:** Knut Hestad, Knut Engedal, Peter Horndalsveen, Bjørn Heine Strand

**Affiliations:** ^1^Department of Health- and Nursing Science, Faculty of Social and Health Sciences, Inland Norway University of Applied Sciences, Elverum, Norway; ^2^Department of Research, Innlandet Hospital Trust, Ottestad, Norway; ^3^Norwegian National Advisory Unit on Ageing and Health, Vestfold County Hospital Trust, Tønsberg, Norway; ^4^Department of Geriatric Medicine, Oslo University Hospital, Oslo, Norway; ^5^Department of Old Age Psychiatry, Innlandet Hospital Trust, Ottestad, Norway; ^6^Department of Chronic Diseases and Ageing, Norwegian Institute of Public Health, Oslo, Norway

**Keywords:** blood pressure, Alzheimer’s disease, aging, mild cognitive impairment, subjective cognitive decline

## Abstract

The aim of the study was to investigate whether blood pressure (BP) differed among people with different dementia diagnoses, mild cognitive impairment, and subjective cognitive decline and whether BP differences were observed across age and sex. Our study population comprised clinical data from 6,236 patients (53.5% women) aged 45–97 years (*Mean* = 73.9, *SD* = 9.6) referred to dementia assessment in 42 outpatient clinics across Norway during 2009–2019. Patients with the following diagnoses were included: Subjective cognitive decline (SCD), Mild cognitive impairment (MCI), dementia due to Alzheimer’s disease (AD), Vascular dementia (VaD), mixed AD and VaD, and dementia in Parkinson’s disease/Lewy body disease (PDD/LBD). For all diagnostic groups, SBP increased with age until about 80 years, after which it trended downward, whereas DBP declined after 60 years of age for all diagnostic groups. Patients aged 65 years and younger with SCD had lower SBP compared to AD patients at the same age, but SBP increased rapidly with increasing age, resulting in a substantially higher SBP at 80 + years compared with all other diagnostic groups. No other differences in SBP or diastolic blood pressure (DBP) were found among patients with the different dementia diagnosis. Neither SBP nor DBP differed between MCI and AD groups. An interaction between age and gender was found for SBP at younger ages, as women started out with a lower pressure than men did but ended up with higher SBP.

**Conclusion:**

Among 80+ patients, blood pressure did not differ as a function of the various dementia disorders. The SBP for the SCD patients of various age groups differed from all other diagnostic groups, indicating either that internal regulation of BP in older people is a risk factor for dementia or that brain damage causing dementia or MCI may led to changes in blood pressure. Brain aging seems to influence SBP differently in men and women.

## Introduction

It is well established that hypertension in midlife is a risk factor for vascular dementia (VaD) and dementia due to Alzheimer’s disease (AD) (Launer et al., 1995; Swan et al., 1998a, b; Kilander et al., 1998; Kivipelto et al., 2001; Yamada et al., 2003; Whitmer et al., 2005; Kimm et al., 2011; Joas et al., 2012; Livingston et al., 2017). In addition, hypertension is the most prominent risk factor for stroke. Although various researchers consider VaD and AD to be two conditions with different pathological mechanisms, a growing number of studies suggests that the co-occurrence of mixed dementias of these two types (i.e., VaD and AD) are common among the oldest old (Ihle-Hansen et al., 2012; Livingston et al., 2017). Dementia that occurs after a stroke, often called post-stroke dementia, may be due a mixture of AD and brain damage caused by the stroke (Ihle-Hansen et al., 2011; Ihle-Hansen et al., 2012; Ashraf et al., 2016). Even though there is a strong association between dementia and high blood pressure (BP) in midlife, the age at hypertension onset seems to be a key factor in that association (Livingston et al., 2017). Whether elevated BP with late-life onset is a risk factor for the development of dementia is uncertain. One study found that midlife, but not late-life, elevated systolic BP (SBP) was related to cognitive decline (Gottesman et al., 2014). In another study, midlife SBP as low as 130 mmHg was associated with increased risk of dementia, whereas no such association was found for SBP with late-life onset (Livingston et al., 2017; Abell et al., 2018). Yet a previous study found no association between elevated BP and cognitive decline in older individuals (Hebert et al., 2004). Most studies do conclude, however, that midlife elevated SBP is strongly associated with the development of dementia and MCI in later life (Gottesman, 2019).

The association between high SBP and dementia is reported to be diminished immediately prior to or close to the diagnosis of dementia (Joas et al., 2012) and as early as 1996, Skoog et al. (1996) determined that SBP declined after the diagnosis of both AD and VaD. In their interpretation of the data, Skoog et al. said that the extent to which decline in BP before dementia onset is a cause or consequence of brain disease remains to be elucidated. Most of the literature on BP in people with dementia has reported that high BP is a key risk factor in white matter changes (WMC) and that WMC could possibly cause changes in BP (Liao et al., 1996; Basile et al., 2006; Nasrallah et al., 2019). Knowledge about the development of BP in people with MCI and dementia is key from a treatment perspective. In two articles, Gottesman has discussed antihypertensive treatment to prevent the development of dementia (Gottesman, 2018, 2019), and Williamson et al. (2019) have suggested that aggressive antihypertensive treatment could reduce the incidence of MCI and dementia, as shown in some clinical trials (Peters et al., 2019). Nasrallah et al. have demonstrated that BP treatment may attenuate white matter hyperintensities and total brain volumes (Nasrallah et al., 2019). In addition, Shah et al. have reported that elevated midlife BP may be a compromising vascular integrity, leading to cerebral amyloid angiopathy and impaired amyloid beta-protein (Aβ) clearance from the brain, thus indicating that the Aβ-related risk for AD was higher when BP was higher (Shah et al., 2012).

Various authors have associated low SBP with cognitive impairment and dementia in persons over 80 years of age (Verghese et al., 2003; Hestad et al., 2005; Euser et al., 2009; Gabin et al., 2017). It is also known that hypoperfusion is related to both blood-brain-barrier (BBB) impairment and white matter (and normal-appearing white matter) hyperintensities (Wong et al., 2019). From the Swedish Kungsholmen project, which included people 75 years and older, a reduction in SBP with at least 10 mm hg from baseline to follow-up was associated with cognitive decline independent of antihypertensive medication use (Zhu et al., 1998). Ruitenberg et al. (2001) have suggested that there is an inverse association between BP and dementia risk in elderly persons on antihypertensive medication. It may be that older people need higher BP in order to obtain adequate cerebral perfusion or that their lower BP could be secondary to brain lesions in preclinical stages of dementia (Ruitenberg et al., 2001). Furthermore, Heijer et al. (2003) have shown that both high and declining diastolic BP (DBP) are associated with global brain atrophy, as indicated by magnetic resonance imaging (MRI). In addition, dysregulation of BP may play a significant role in the neural integrity of old people, which could lead to cerebrovascular pathology, especially in the frontal and parietal white matter (Salat et al., 2012). Both high and low BP may be related to the brain’s difficulty in compensating for large changes in pressure. The degenerative process may start with hypertension, but in the long run BBB disturbances and white matter disease may result in low BP.

With regard to the association between BP for various dementia disorders, the literature is scarce. As indicated here, there are strong associations between dementia of AD, white matter lesions and VaD, but less is known about any possible influence of PDD/LBD on BP. It is known, however, that the regulation of BP is often disrupted in PDD/LBD. We assume, therefore, that the association between BP and dementia due to these two disorders differ from the association between BP on the one hand and AD and VaD on the other (Velseboer et al., 2011; Espay et al., 2016; Udow et al., 2016). One study reported that SBP is lower in LBD than it is in AD (Chan et al., 2018).

Regarding BP regulation in healthy old people, the trajectory of increasing BP differs in men and women as they age (Joyner et al., 2016). Joyner et al. (2016) suggested that this difference is related to their different physiological control of BP. The β-adrenergic vasodilator mechanisms are diminished in women during the aging process, whereupon stronger muscle sympathetic nerve activity is seen. Thus, the relationship between sympathetic muscle nerve activities shows a positive association with BP, which is stronger in women than in men. There also seems to be a different incidence of myocardial infarction for men and women, at least until 95 years of age, which could support this difference in sympathetic nerve activity between old men and old women (Barrett-Connor, 2013; Daka et al., 2015). Daka et al. (2015) suggest that the lower incidence rates for women may be caused by women and men’s different endothelin-1 levels.

In summary, most studies have examined how blood pressure may precede dementia in general and AD in particular, but there is little knowledge regarding BP levels at different ages in patients with dementia. Given this background, there is a need to know how BP is distributed across ages in the various dementia diagnoses, MCI, and SCD.

## Hypothesis

There is an association between midlife hypertension and late-life hypotension together with dementia incidents, which seems to be more strongly associated with incident dementia prior to 74 years of age (Walker et al., 2019). Given the reported association between high SBP and dementia, which seems to flatten out just before or close to the diagnosis of dementia (Skoog et al., 1996; Joas et al., 2012), or even decline, our hypothesis is that patients with a diagnosis of AD or VaD will have a lower SBP than those with subjective cognitive decline (SCD). We further hypothesized that people with PDD/LBD will have different SBPs than will those with AD or VaD, and that that men and women will demonstrate different trends in SBP across age groups.

## Aims

The aim of this study was to investigate whether BP differed between dementia diagnoses and milder forms of impaired cognition, and whether these differences were observed across age and gender.

## Materials and Methods

Our study population consisted of clinical data from 6,236 patients (53.5% women) aged 45–97 years (*Mean* = 73.9, *SD* = 9.6) referred to dementia assessment in one of 42 outpatient clinics across Norway during 2009–2019 and included in the Norwegian Register of Persons Assessed for Cognitive Symptoms (NorCog) (Naavik et al., 2017).

## The NorCog Register

NorCog is a national research and quality register for persons referred to the specialist health care service for the study of memory impairment and possible dementia. The register is consent-based and has existed since 2009; 42 outpatient clinics currently collect data for the registry on regular patients visiting the clinics. Data from more than 10,000 patients are found in the registry, which is licensed by the Norwegian Data Inspectorate until 2030. Of those patients asked to give consent, 90% have answered positively and signed a consent form. The data collected in the register include a wide battery of neuropsychological tests, a comprehensive physical examination, blood sample collection for various types of analyses, and cerebral imaging with MRI or CT (Braekhus et al., 2011). In addition, and in accordance with the Norwegian national guidelines on dementia, fluorodeoxyglucose-positron emission tomography (FDG-PET) and examination of concentration of amyloid-beta and tau protein in cerebrospinal fluid are increasingly performed in many patients with an uncertain diagnosis^1^.

## Diagnoses

The dementia diagnoses in NorCog are based on the International Classification of Diseases (ICD-10) criteria for research (WHO, 1993) and made by experienced neurologists, geriatricians, or geriatric psychiatrists. The Winblad criteria are used for MCI (Winblad et al., 2004), and patients who complained about cognitive decline but showed no cognitive impairment are given the diagnosis of SCD (Jessen et al., 2014). Patients with the following diagnoses were included in the analyses: SCD, MCI, AD, VaD, mixed AD and VaD, and PDD/LBD. Patients with Frontotemporal dementia (FTD) and unspecific dementia or dementia due to rare causes were excluded because of small samples. Among patients diagnosed with AD, 138 with a history of stroke were removed. All patients were evaluated with magnetic resonance imaging (MRI) or computer tomography (CT) caput as part of the standardized diagnoses work up (Braekhus et al., 2011). We examined how many of the participants included between 2009 and 2014 were prescribed various drugs. In our population 13.3% used antidementia medication, 5.4% were on Antidiabetic medication, 13.7% used antidepressants, 1.5% used antipsychotics, and 8.7% used tranquilizers (benzodiazepines or hypnotics).

## Blood Pressure

Nurses measured SBP and DBP in a clinical setting, after the seated patients had become comfortable with the situation – about 30 min into the consultation. If BP was evaluated as high, if the patient seemed to be stressed, or if there appeared to be a white-coat syndrome, BP was measured again. The last measurement was then entered into the patient’s record and the register. Regarding BP regulation, antihypertensive medications were not in the register the first years. Thus, data on antihypertensive medications were available for a subsample.

## Mini-Mental State Examination (MMSE)

We used the Norwegian-validated MMSE version as a measure of severity of global cognition (Folstein et al., 1975; Engedal et al., 1989). Patients with a diagnosis of SCD with an MMSE score below 25 (*n* = 137) or MCI with MMSE score below 18 (*n* = 56) were regarded as having an uncertain diagnosis and were therefore not included in the analyses.

## Statistical Analyses

We applied standard methods in our analyses to investigate differences in BP among patients with different cognitive disorder diagnoses. First, we applied one-way ANOVA to test for differences in mean BP between diagnose groups. Second, to account for potential confounding by age and gender, we applied linear regression. All interactions among age, sex, and diagnoses were investigated. Third, to model the non-linear age trend in BP, age was modeled as a cubic spline in a multivariable regression spline model (mvrs package in Stata, developed by Patrick Royston, MRC Clinical Trials Unit, London, United Kingdom). In these non-linear analyses, we observed a strong shift in SBP at around 80 years of age, and therefore we did a stratified analysis in two age strata (<80 years, ≥80 years), and analyzed differences in BP between diagnoses with AD as reference diagnosis in each age stratum, applying age- and sex-adjusted linear regression.

### Ethics Statement

The study was approved by the Regional ethics committee for medical and biological research (REK: 2019/316), and all participants gave written informed consent.

## Results

MCI was the most prevalent diagnosis (40%), followed by AD (29%), and SCD (13%). Mixed AD/VaD (8%), VaD (6%), and PDD/LBD (5%) were less prevalent (Table 1).

**TABLE 1:** Mean (SD) blood pressure (mmHg) across age groups and diagnoses.

	Age < 65 years		Age 65–74 years		Age 75–79 years		Age 80–84 years		Age 85 + years	
					
	Mean systolic BP, mmHg (SD)	*n*	Mean systolic BP, mmHg (SD)	*n*	Mean systolic BP, mmHg (SD)	*n*	Mean systolic BP, mmHg (SD)	*n*	Mean systolic BP, mmHg (SD)	*n*
SCD	137.3 (17.9)	352	146.1 (20.5)	245	150.1 (25.4)	100	158.4 (25.5)	54	152.4 (23.9)	41
MCI	140.9 (19.7)	469	145.4 (21.0)	831	149.3 (22.8)	533	146.7 (22.6)	415	146.4 (23.5)	248
AD	140.4 (20.4)	202	147.0 (20.8)	512	149.5 (23.0)	448	150.0 (22.5)	361	146.3 (23.6)	275
Mixed AD/VaD	147.1 (5.8)	8	148.4 (24.3)	103	147.6 (20.3)	135	149.0 (21.6)	140	145.6 (24.0)	105
VaD	143.7 (20.8)	15	151.3 (24.8)	78	142.1 (22.1)	97	148.3 (21.4)	106	149.5 (29.8)	78
PDD/LBD	146.7 (20.9)	27	142.9 (21.9)	116	143.6 (20.1)	64	145.4 (27.3)	50	141.8 (26.3)	28
*p*-value*	0.02		0.04		0.02		0.01		0.4	

	**Mean diastolic BP, mmHg (SD)**	**n**	**Mean diastolic BP, mmHg (SD)**	**n**	**Mean diastolic BP, mmHg (SD)**	**n**	**Mean diastolic BP, mmHg (SD)**	**n**	**Mean diastolic BP, mmHg (SD)**	***n***

SCD	83.6 (10.6)	352	84.4 (12.6)	245	82.0 (13.1)	100	83.1 (12.3)	54	82.0 (12.1)	41
MCI	85.6 (11.0)	469	83.5 (12.1)	831	81.1 (12.6)	533	80.0 (11.8)	415	77.5 (12.6)	248
AD	84.0 (11.1)	202	81.8 (10.9)	512	82.5 (11.6)	448	80.6 (12.0)	361	78.6 (12.3)	275
Mixed AD/VaD	88.8 (9.6)	8	83.8 (14.4)	103	80.6 (12.9)	135	78.5 (12.5)	140	76.0 (13.9)	105
VaD	86.5 (13.8)	15	86.1 (15.5)	78	80.6 (12.0)	97	79.6 (12.3)	106	79.5 (17.0)	78
PDD/LBD	88.4 (11.0)	27	82.8 (11.4)	116	80.3 (12.5)	64	80.3 (10.2)	50	74.6 (11.9)	28
*p*-value*	0.03		0.03		0.39		0.27		0.09	

*One-way ANOVA, prob > F. SCD, Subjective cognitive decline; MCI, mild cognitive impairment; AD, Dementia of Alzheimer’s disease; Mixed AD/VaD, combination of Alzheimer’s disease and Vascular dementia; VaD, Vascular dementia; PDD/LBD, combination of dementia in Parkinson’s disease and Lewy body disease; BP, blood pressure.

Those patients with SCD were younger on average (*mean* = 66.5 years, SD 10.5) than were patients of the other groups: MCI (73.0, SD 9.4), AD (75.7, SD 8.6), Mixed AD/VaD (79.2, SD 6.7), VaD (78.5, SD 6.9), PDD/LBD (74.5, SD 7.7). As expected, the MMSE scores were lower for the patients with dementia than for patients with MCI and SCD. Mean MMSE score was 20.8 for AD and Mixed AD/VaD, 21.3 for VaD, 21.8 for PDD/LBD, whereas it was 25.7 for MCI and 28.4 for SCD.

### Blood Pressure by Diagnosis

Among the patients below 80 years of age, BP did not differ significantly between the diagnostic groups (Table 2).

**TABLE 2:** Differences (95% confidence interval, CI) in mean blood pressure between diagnoses, stratified by age.

Diagnosis				

	Systolic bp (mmHg)			
	
(Age < 80/Age ≥ 80)	Mean absolute difference in BP (95% CI) Age < 80 years	*p*-value	Mean absolute difference in BP (95% CI) Age ≥ 80 years	*p*-value
SCD (697/95)	−1.26 (−3.35, 0.83)	0.24	7.72 (2.55, 12.69)	< 0.01
MCI (1833/663)	−0.58 (−2.16, 1.00)	0.47	−1.87 (−4.42, 0.70)	0.15
AD (1162/636)	REF	–	REF	–
Mixed AD/VaD (246/245)	−0.14 (−3.01, 2.80)	0.93	−0.79 (−4.24, 2.66)	0.65
VaD (190/184)	−1.61 (−4.90, 1.68)	0.21	0.40 (−3.44, 4.25)	0.64
DLB/PDD (207/78)	−3.03 (−6.20, 0.14)	0.06	−4.31 (−9.82, 1.19)	0.13
Overall p-value*	*0.32*		< *0.01*	

	**Diastolic bp (mmHg)**			

SCD (697/95)	0.08 (−1.09, 1.25)	0.89	2.66 (−0.04, 5.35)	0.05
MCI (1833/663)	0.44 (−0.44, 1.33)	0.33	−0.93 (−2.29, 0.43)	0.18
AD (1162/636)	REF	–	REF	–
Mixed (246/245)	0.10 (−1.55, 1.75)	0.91	−2.23 (−4.07, −0.39)	0.02
VaD (190/184)	1.00 (−0.83, 2.85)	0.28	−0.37 (−2.42, 1.68)	0.72
DLB/PDD (207/78)	0.03 (−1.75, 1.80)	0.98	−1.60 (−4.53, 1.32)	0.28
Overall p-value*	*0.85*		*0.02*	

Adjusted by age and sex in linear regression. *Likelihood ratio test. SCD, Subjective cognitive decline; MCI, mild cognitive impairment; AD, Dementia of Alzheimer’s disease; Mixed AD/VaD, combination of Alzheimer’s disease and Vascular dementia; VaD, Vascular dementia; PDD/LBD, combination of dementia in Parkinson’s disease and Lewy body disease; BP, blood pressure; CI, confidence interval.

In the 80 + age stratum, however, the SCD group had elevated BP compared with the other diagnostic groups; 7.7 mmHg higher mean SBP compared with the AD group (*p* < 0.01), and 2.7 mmHg higher DBP than in AD patients (*p* = 0.05). Furthermore, those with mixed type of AD/VaD had 2.2 mmHg lower DBP than AD patients (*p* = 0.02). We did an additional sensitivity analysis, excluding all patients with a history of stroke, and this analysis did not change the results significantly (data not shown).

### Blood Pressure by Age

**FIGURE 1:**
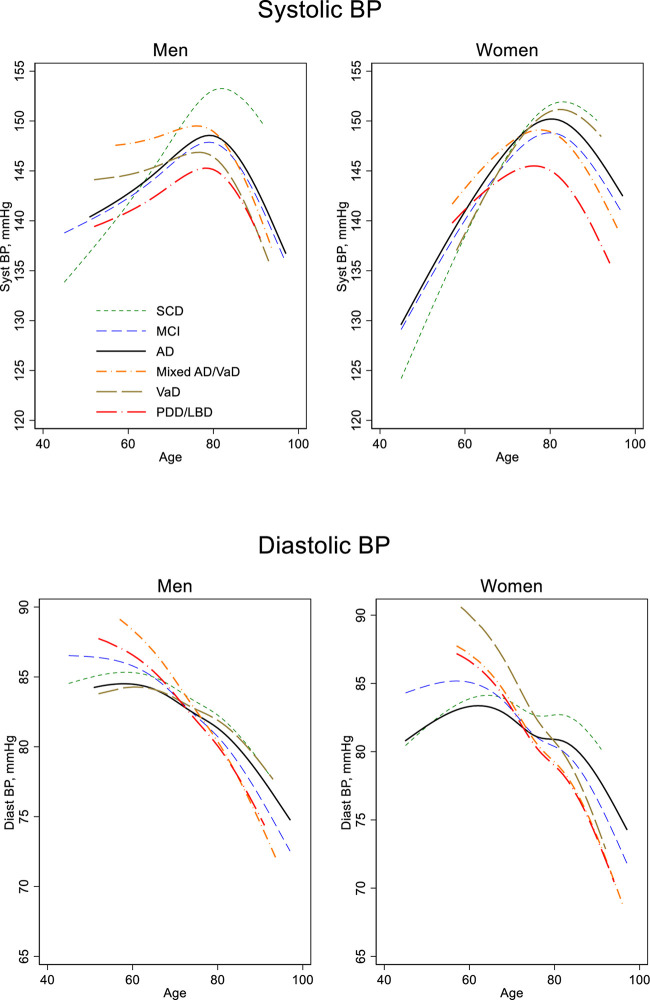
Blood pressure by age, sex, and diagnosis. Estimated in a regression spline (RS) model with age modeled as a cubic spline and including the interaction diagnosis by age. SCD, Subjective cognitive decline; MCI, mild cognitive impairment; AD, Dementia of Alzheimer’s disease; Mixed AD/VaD, combination of Alzheimer’s disease and Vascular dementia; VaD, Vascular dementia; PDD/LBD, combination of dementia in Parkinson’s disease and Lewy body disease; BP, blood pressure.

When examining BP by age, it is important to remember that we did not have repeated measures; because we did not follow individuals longitudinally, the associations reported in this paper are merely cross-sectional. In general, BP was associated with age, but the pattern differed between SBP and DBP. DBP declined monotonously with age, at least from age 60 years, whereas SBP increased with age up to around 80 years and then declined, following an n-shaped curve (Table 1 and Figure 1).

### Age Trends in Blood Pressure: Sex Matters, but Only Before the Age of 80

Below 80 years of age, the age trends in SBP differed significantly between men and women. Women had 6.0 mmHg lower SBP at age 50 years than men, *p* < 0.001, but the increase with age was larger in women (6.0 vs. 3.1 mmHg per 10 years, *p* < 0.001). This difference in age trends by sex can be visually observed in Figure 1, in which steeper slopes occur before age 80 years in women compared to the slopes in men. The slope differences between men and women did occur for all diagnose groups. After the age of 80 years, however, age trends in BP were similar in men and women.

### Age Trends in Blood Pressure: Diagnosis Matters, but Only Before the Age of 80 in Men

For DBP before age 80 years, patients with AD had a less rapid drop compared with the patients of the other dementia diagnostic groups (Figure 1). Age trends for MCI and SCD, however, did not differ from that of AD. For SBP the age trends were similar in women. But in men, those with VaD (*p* = 0.03) or Mixed AD/VaD (*p* = 0.07) had a less rapid increase with age compared to those with an AD diagnosis. After the age of 80 years, the age trends in BP (SBP and DBP) did not differ between any of the diagnose groups.

Of the 6,236 participants, we had valid information on use of BP medications in 4,646. Controlling for this information in the analysis did not change the differences between the diagnostic groups or conclusions (data not shown).

## Discussion

For all diagnostic groups, SBP increased with age until about 80 years, after which a downward trend occurred; whereas, for DBP, a monotonous decline with age was observed for all diagnostic groups from about age 60. Thus, BP for the groups studied did not follow a linear trend. Rather, as can be seen in Figure 1, the trend varied somewhat among the groups representing the age at which the BP slope turned, and how much the slopes increased or decreased among the diagnostic groups. We found differences among diagnostic groups in SBP level, depending on age at SBP measurement.

The patients with PDD/LBD had lower SBP at younger ages than AD patients did, but they did not differ from the other groups among patients 80 and older. Patients with SCD had a lower SBP at younger ages compared to AD patients, but SBP increased rapidly with increasing age, resulting in a substantially higher SBP at older ages for the SCD compared with the other diagnostic groups.

The Lancet Commission on Hypertension states that if we live long enough, hypertension is an inevitable consequence of aging, even for those people who reach middle age without hypertension. Middle-aged people without hypertension will have a greater than “90% chance” of developing high BP during their remaining lifetime (Olsen et al., 2016; O’Brien, 2017). Nevertheless, some studies have not found SBP to increase with age in the healthy elderly (Hopstock et al., 2015; Holmen et al., 2016). Hopstock et al. reported, however, that there were some limitations related to selection bias in their study, the most significant bias being that 40% of subjects had only one BP measurement. Our results, in people with SCD are in line with the findings of the Lancet Commission, examining SBP before the age of 80. We did find, however, that people aged 80 + had lower SBP than did people < 80, and similar results were found in DBP for people aged 60 + vs. those < 60 years of age. It may be that between 60 and 80 years there is a unilaterally increase in SBP, or it may be that people visiting a memory clinic for subjective cognitive decline are not representative of the general population. All SCD patients included in the study had approached the clinics for evaluation of memory problems or various types of dementia.

The MCI and the AD groups showed highly similar BP development, perhaps because of the large conversion rate from MCI to dementia, especially to AD. Various authors have reported that up to 50% of all people with a MCI diagnosis will develop dementia during a 5-year follow-up (Visser et al., 2006; Mauri et al., 2012). Thus, many of our MCI patients may have preclinical AD in which elevated SBP is likely to flatten out (Joas et al., 2012). We were not surprised to see that the PDD/LBD group had the lowest SBP among the dementia groups, as this finding is in accordance with our hypothesis and supported by the Chan et al. (2018) study. BP regulation in Parkinson’s and diffuse Lewy body disease with and without dementia are probably disrupted due to damage in brain areas responsible for automatic biological regulation systems (Velseboer et al., 2011; Espay et al., 2016; Udow et al., 2016).

Regardless of their etiology, people with dementia had different SBP across age groups than did people with SCD. As suggested by others, brain damage causing dementia may also cause dysfunction in BP regulation. We cannot explain why BP is lower in the oldest people with dementia compared to those with no cognitive impairment (the SCD group). The results in our study is in line with other studies (Skoog et al., 1996; Skoog and Gustafson, 2003) and could explain the varying results of studies reporting elevated BP as risk factors for dementia in old age (Hebert et al., 2004; Gottesman et al., 2014; Abell et al., 2018). If future studies could confirm that BP drops by development of dementia, it would have consequences for clinical practice. BP treatment should be suggested or even withdrawn in patients with dementia showing declining BP.

We found an interaction between age and sex for SBP at younger ages, in that women start out with a lower pressure but end up with a higher blood pressure than men. It is seen from population studies that men and women show differential developmental trajectories regarding BP regulations over a lifetime (Joyner et al., 2016; Hestad et al., 2020). In the present study there were significant differences between men and women in blood pressure development. It could be that the decline in SBP observed in 80 + patients is related to a terminal drop – that some vital functions decline as part of the aging process (Delgado et al., 2018; Goodwin, 2018).

One strength of this study is the large number of participants – 90% of the entire population of patients referred for dementia assessment in the Norwegian specialist health service between 2009 and 2019. The study has some limitations, however. The design is cross-sectional, which means that our results describing the relationships between BP and age should be interpreted with caution. BP was measured by various nurses using different BP devices, and BP was measured at only one time. It is suggested, however, that variability in blood pressure over time may be harmful to the brain and related to dementia development (Yoo et al., 2020) and Lattanzi et al. (2015) even suggested that BP variability may play different roles according to stage and types of dementia (Lattanzi et al., 2015). We had valid BP medication data for only a subsample of the patients (4,646 of 6,377), which could have influenced the results. In addition, some antihypertensive drugs may protect the brain more than others from damage related to BP oscillations (Hestad and Engedal, 2006; Tully et al., 2016; Yoo et al., 2020).

## Conclusion

For 80 + patients, BP did not differ among people with various dementia disorders, but PDD/LBD patients under age 80 had lower SBP than the AD patients. Furthermore, those with SCD had higher BP (both SBP and DBP) at older ages than all other diagnostic groups, indicating either that regulation of BP in older people poses a risk factor for dementia or that brain damage causing dementia or MCI may lead to changes in BP. Brain aging seems to influence SBP differently in the older men and women.

## Data Availability Statement

The data analyzed in this study was subjected to the following licenses/restrictions: The data was collected from the Norwegian Register of Persons Assessed for Cognitive Symptoms (NorCog). The data can be available after approvement from the board of the database. Requests to access these datasets should be directed to Marit Nåvik, naam@sthf.no.

## Ethics Statement

The studies involving human participants were reviewed and approved by the study Regional Ethics Committee for Medical and Biological Research (REK: 2019/316), and all participants gave written informed consent. The patients/participants provided their written informed consent to participate in this study.

## Author Contributions

KH initiated and planned the study. PH and KE took part in the planning of the study. BS did all the statistical analyzes. KH and BS wrote the first draft of the manuscript. All authors took part in the writing process.

## Conflict of Interest

The authors declare that the research was conducted in the absence of any commercial or financial relationships that could be construed as a potential conflict of interest.

## Footnotes

1 https://www.helsedirektoratet.no/retningslinjer/demens
